# **Machine learning identifies abnormal Ca**^**2**+^**transients in human induced pluripotent stem cell-derived cardiomyocytes**

**DOI:** 10.1038/s41598-020-73801-x

**Published:** 2020-10-12

**Authors:** Hyun Hwang, Rui Liu, Joshua T. Maxwell, Jingjing Yang, Chunhui Xu

**Affiliations:** 1 Division of Pediatric Cardiology, Department of Pediatrics, Emory University School of Medicine and Children’s Healthcare of Atlanta, Atlanta, GA 30322 USA; 2 Department of Pediatrics, The Third Xiangya Hospital of Central South University, Changsha, 410013 Hunan China; 3 Center for Computational and Quantitative Genetics, Department of Human Genetics, Emory University School of Medicine, Atlanta, GA 30322 USA; 4 Wallace H. Coulter Department of Biomedical Engineering, Georgia Institute of Technology and Emory University, Atlanta, GA 30033 USA

**Keywords:** Machine learning, Induced pluripotent stem cells

## Abstract

Human-induced pluripotent stem cell-derived cardiomyocytes (hiPSC-CMs) provide an excellent platform for potential clinical and research applications. Identifying abnormal Ca^2+^ transients is crucial for evaluating cardiomyocyte function that requires labor-intensive manual effort. Therefore, we develop an analytical pipeline for automatic assessment of Ca^2+^ transient abnormality, by employing advanced machine learning methods together with an Analytical Algorithm. First, we adapt an existing Analytical Algorithm to identify Ca^2+^ transient peaks and determine peak abnormality based on quantified peak characteristics. Second, we train a peak-level Support Vector Machine (SVM) classifier by using human-expert assessment of peak abnormality as outcome and profiled peak variables as predictive features. Third, we train another cell-level SVM classifier by using human-expert assessment of cell abnormality as outcome and quantified cell-level variables as predictive features. This cell-level SVM classifier can be used to assess additional Ca^2+^ transient signals. By applying this pipeline to our Ca^2+^ transient data, we trained a cell-level SVM classifier using 200 cells as training data, then tested its accuracy in an independent dataset of 54 cells. As a result, we obtained 88% training accuracy and 87% test accuracy. Further, we provide a free R package to implement our pipeline for high-throughput CM Ca^2+^ analysis.

## Introduction

Cardiomyocytes derived from human-induced pluripotent stem cells (hiPSC-CMs) are highly desired for drug discovery and modeling human development and disease, as alternative models such as human primary CMs are hard to obtain^1^. Although current hiPSC-CMs display fetal-like phenotypes in terms of their structural and electrophysiological properties^2^, they have increasingly been used to study normal cardiac functionality^3–5^ and human cardiovascular diseases such as long QT syndrome, catecholaminergic polymorphic ventricular tachycardia and viral myocarditis as well as for high-throughput cardiotoxicity screening^5–9^. Furthermore, hiPSC-CMs are under active investigation for use as a cell source for possible clinical usage^10,11^. For these applications, extensive functional characterization of hiPSC-CMs is required.

Ca^2+^ transients are a fundamental characteristic of cardiomyocyte functionality, although cardiac action potentials and contractility are also commonly used to study cardiomyocyte functionality by methods such as patch clamp, multielectrode array, microscopic video analysis, and fluorescence imaging^12^. Coordinated movement of Ca^2+^ at single cell level plays a key role to control contraction of the heart by the conversion of electric excitation into mechanical contraction. Specifically, each action potential induces Ca^2+^ influx, which triggers a much greater Ca^2+^ release from the sarcoplasmic reticulum (SR). The increased cytosolic Ca^2+^ binds to and activates the Ca^2+^-sensing protein of the contractile apparatus and initiates CM contraction. Then Ca^2+^ is removed from the cytosol through reuptake into the SR or extrusion into the extracellular space, which leads to CM relaxation. Thus, the rapid release and reuptake of Ca^2+^ between the SR and the cytosol create a Ca^2+^ transient inside the CM^13^. Abnormal Ca^2+^ signals are indicative of various cardiac pathologies, such as arrhythmia^5,6,8^.

An accurate Ca^2+^ transient analysis is an important component of hiPSC-CM phenotype analysis. Ca^2+^ transient characteristics are commonly captured with Ca^2+^-specific fluorescent dye, and fluorescence imaging is the most optimal for high-throughput application. Human experts’ assessment of Ca^2+^ transient signals is often based on Ca^2+^ transient morphology characterized by rapid upstroke and decay kinetics. Although manual identification of abnormal Ca^2+^ transients by human experts is often taken as gold standards, such visual assessment is labor-intensive, time-consuming, and subjective to the assessor’s expertise. Moreover, manual identification of abnormal Ca^2+^ transients by human experts becomes a bottleneck hindering its application to high-throughput analysis. Thus, a user-friendly computational tool is in pressing need to mitigate the bottleneck of manual analysis and to enable automatic assessment of Ca^2+^ transient abnormality.

Previously, Juhola et al*.* proposed an Analytical Algorithm to detect cycling Ca^2+^ transient peaks, quantify peak variables, and assess the abnormality of transient peaks and signals^14^. This analytical algorithm identifies signal abnormality based on whether the assessed cell signal contains at least one abnormal transient peak based solely on characteristics of a single peak. The assessment did not leverage shared characteristics of normal and abnormal Ca^2+^ transient peaks and signals across all samples, which are expected to provide valuable input to improve the accuracy of signal abnormality assessment. Further, the analytical algorithm fails to account for the valuable manual assessment results about existing data.

To overcome these limitations, we develop an improved automatic pipeline that is composed of peak detection, peak variable quantification, peak abnormality assessment, signal variable extraction, and signal abnormality assessment. We adapt the existing Analytical Algorithm for peak detection, peak variable quantification, and peak abnormality assessment. Additionally, the advanced machine learning method of Support Vector Machine (SVM)^15^ is used for abnormality assessments of peaks and signals, which leverages shared data characteristics and experts’ manual analysis results of training data.

Further, we provide an R library to implement this pipeline, which includes SVM classifiers trained using our Ca^2+^ transient data as well as functions for peak detection, peak variable quantification, training peak-level SVM classifier, cell variable quantification, training signal-level SVM classifier, and predicting signal abnormality. Our R library is freely available through GitHub and is expected to serve as a convenient tool for people in need of a Ca^2+^ transient analysis software with high speed and accuracy.

## Results

### Study overview

**Figure 1:**
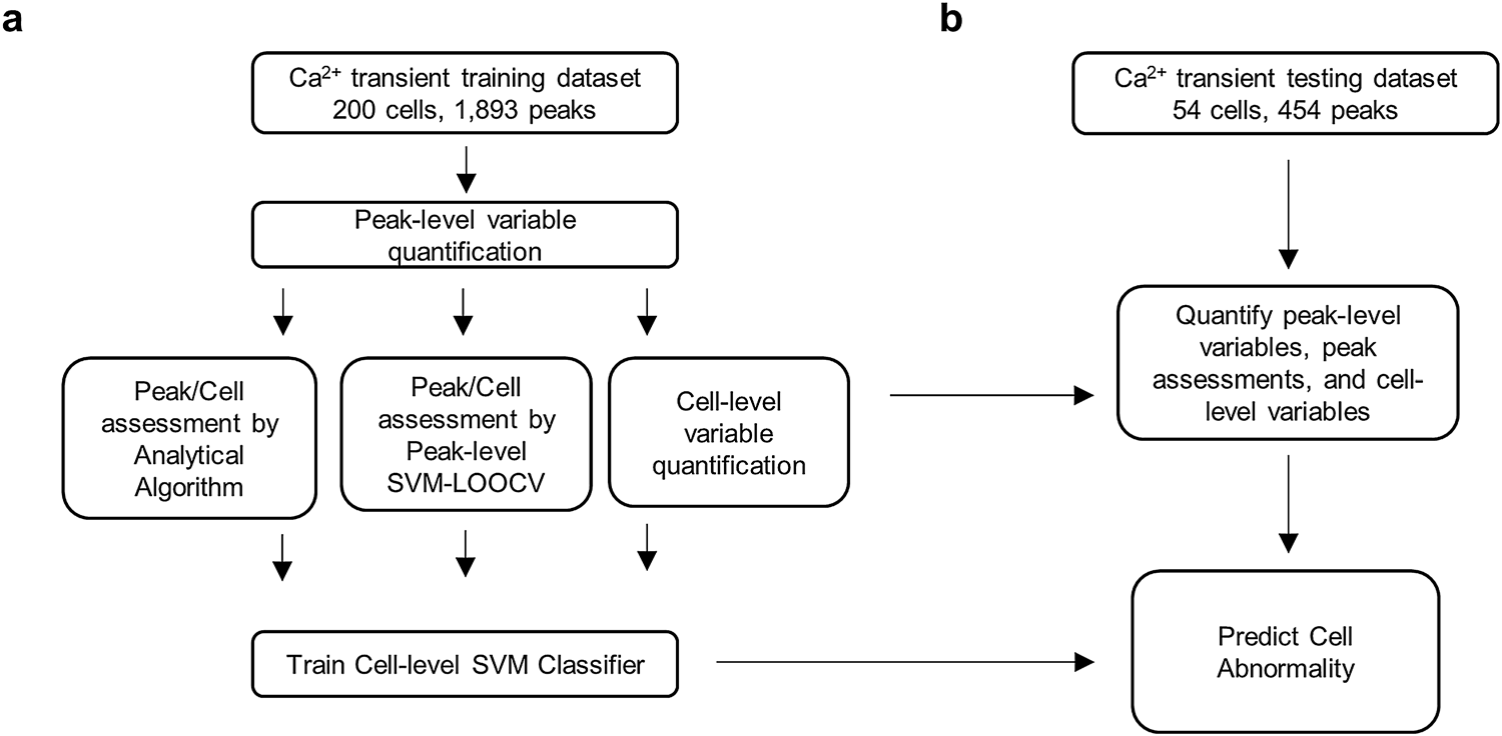
Overall workflow of machine learning method in this study. (**a**) Ca^2+^ transient data of 200 cells and 1893 peaks were collected and analyzed to train the peak- and cell-level SVM models, which were validated via LOOCV. (**b**) Test data of 54 cells and 454 peaks were used to implement the machine learning tool to yield final cell status prediction.

A flowchart is provided in Fig. 1a,b for this pipeline. In this pipeline, we improve the existing Analytical Algorithm^14^ to better characterize signal abnormality by including additional peak variables such as nearby peak distance, varying peak amplitude, and peak asymmetry, as well as considering irregular peak phases for peak abnormality assessment.

In particular, with a set of Ca^2+^ signals as the training data, our pipeline first trains a peak-level SVM classifier by taking peak assessments by human experts as responses (normal or abnormal) and 14 peak variables (the names of the peak variables are listed in Table 1) as predicting features. Second, assessments of peak abnormality by both our improved analytical algorithm and trained peak-level SVM classifiers are obtained and used as additional peak variables. Third, cell abnormality assessment based on those two types of peak assessments along with other cell variables (the names of the cell variables are listed in Table 2) are taken as predictors to train a signal-level SVM classifier for predicting signal abnormality. The trained peak-level and cell-level SVM classifiers can be applied to detect the abnormality of additional Ca^2+^ transient peaks and signals. Additionally, we validated our pipeline using Ca^2+^ transient data generated by our lab.

**Table 1:** Peak variable averages and their standard deviations of the test data.

Peak-level variables	Expert normal n = 372	Expert abnormal n = 82	SVM normal n = 325	SVM abnormal n = 129
A_l	0.04 ± (0.95)	− 0.17 ± (1.18)	0.05 ± (0.98)	− 0.13 ± (1.04)
A_r	0.24 ± (0.90)	− 1.08 ± (0.68)	0.36 ± (0.08)	− 0.91 ± (0.87)
A_d	0.26 ± (0.50)	− 1.19 ± (1.64)	0.40 ± (0.19)	− 1.01 ± (1.42)
D_l	0.09 ± (1.01)	− 0.39 ± (0.83)	0.14 ± (0.99)	− 0.35 ± (0.94)
D_r	0.23 ± (0.94)	− 1.05 ± (0.44)	0.33 ± (0.82)	− 0.84 ± (0.92)
Dy_max	0.03 ± (0.97)	− 0.14 ± (1.14)	0.02 ± (1.02)	− 0.04 ± (0.94)
Dy_min	0.00 ± (0.93)	− 0.01 ± (1.28)	− 0.10 ± (0.88)	0.26 ± (1.22)
D2y_max	− 0.06 ± (0.95)	0.26 ± (1.18)	− 0.05 ± (0.88)	0.12 ± (1.25)
D2y_min	− 0.23 ± (0.68)	1.04 ± (1.46)	− 0.36 ± (0.39)	0.90 ± (1.42)
R	0.20 ± (0.98)	− 0.90 ± (0.40)	0.27 ± (0.94)	− 0.68 ± (0.79)
delta	0.11 ± (1.02)	− 0.50 ± (0.73)	0.10 ± (0.74)	− 0.25 ± (1.44)
delta_l2Dymax	0.06 ± (0.98)	− 0.26 ± (1.05)	0.14 ± (0.99)	− 0.34 ± (0.96)
delta_m2Dymin	0.08 ± (1.04)	− 0.37 ± (0.68)	0.08 ± (1.04)	− 0.20 ± (0.86)
Peak_distance_median	0.12 ± (0.94)	− 0.54 ± (1.09)	0.15 ± (0.71)	− 0.39 ± (1.43)

A 2 × 2 comparison of the peak classification by expert and SVM is shown in Supplementary Table S1 online.

n, number of peaks.

**Table 2:** Cell variable averages and their standard deviations of the test data.

Assessment	Number of cells	Cell-level variables			
		prop_abnormal	var_A	var_delta	var_R
Expert normal	18	− 0.71 ± (0.24)	− 0.48 ± (0.08)	− 0.33 ± (0.01)	− 0.38 ± (0.11)
Expert abnormal	36	0.35 ± (1.05)	0.24 ± (1.15)	0.16 ± (1.20)	0.19 ± (1.18)
SVM normal	19	− 0.81 ± (0.00)	− 0.51 ± (0.04)	− 0.26 ± (0.16)	− 0.43 ± (0.02)
SVM abnormal	35	0.44 ± (1.00)	0.28 ± (1.15)	0.14 ± (1.22)	0.23 ± (1.18)

A 2 × 2 comparison of the cell classification by expert and SVM is shown in Supplementary Table S1 online.

### Data preprocessing

Ca^2+^ transient signal data were generated using MetaXPress software. While the sampling frequency for the transient was 5 Hz across the board, lengths varied between 12 and 32 s. Signals with single peaks were eliminated, as they were insufficient to count as signal data. In particular, we first generated 213 signals: 78 signals from the 12-s dataset and 135 signals from the 32-s dataset. After single-peak signal elimination, 66 from the 12-s dataset and 134 signals from the 32-s dataset were taken as our training dataset. The data were tidied and plotted for assessment by human experts. Human experts labeled peaks and signals as either normal or abnormal. These 200 signals were taken as our training data to validate our proposed pipeline. Following the same procedure, an independent test dataset of 54 cells were generated.

### Abnormality assessment by human experts

**Figure 2:**
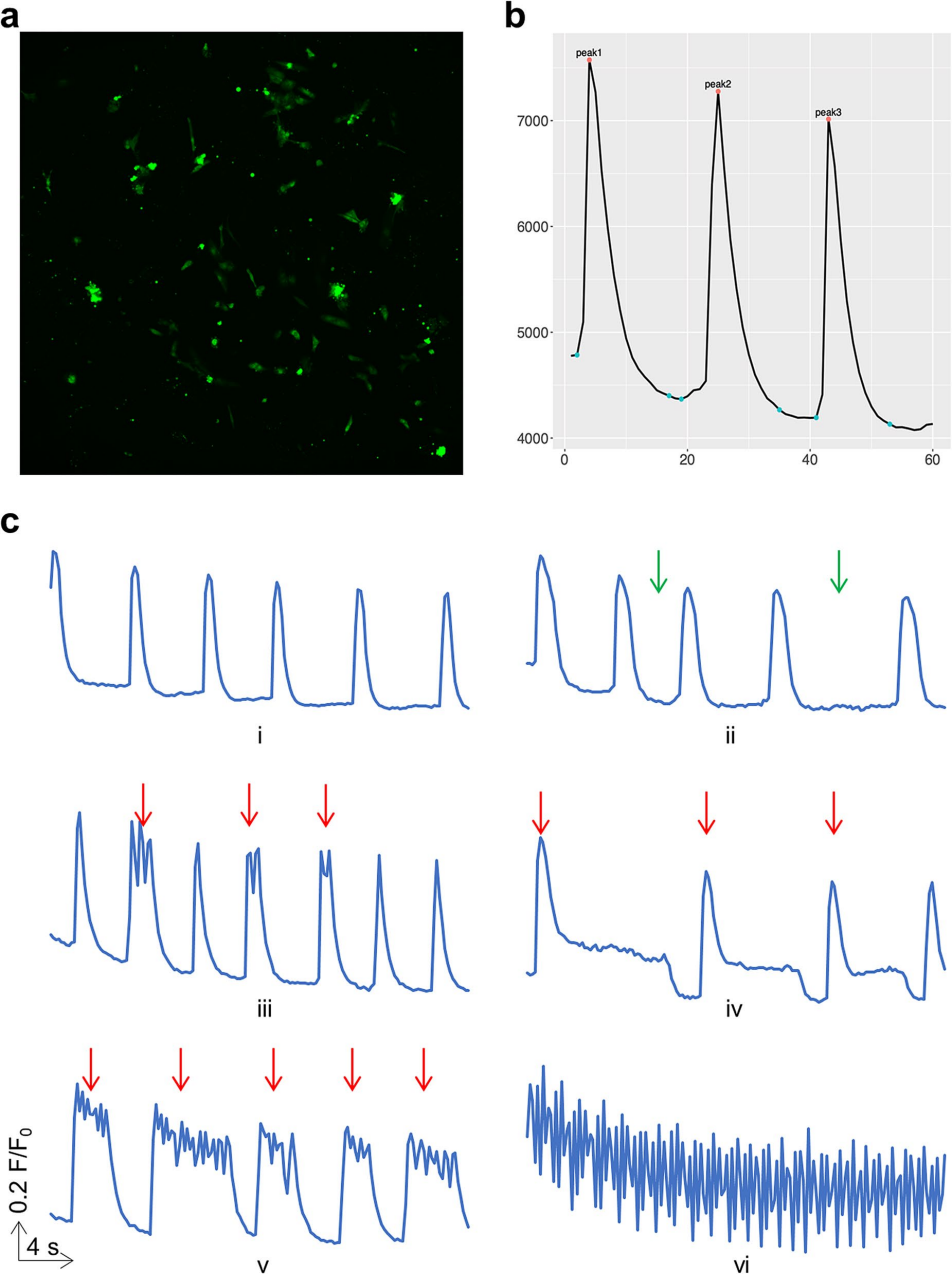
Cells under fluorescence imaging and their peak signals. (**a**) An example of hiPSC-CMs stained with Fluo-4 fluorescing under 488 nm light. (**b**) An example of Ca^2+^ transient signal visualized with detected peaks marked. Number of frames on the x-axis and fluorescence intensity on the y-axis. (**c**) Examples of Ca^2+^ transient signal visualized by human expert. Red arrows denote abnormal peaks and green arrows denote inconsistent periods.

Upon inspection of Ca^2+^ transient signals such as the one shown in Fig. 2a,b, a human expert in assessing Ca^2+^ transient signals made abnormality assessment about the Ca^2+^ transient peaks and signals. For our training dataset, the expert made abnormality assessments for a total of 200 signals and 1893 peaks within those signals. A peak was labeled as normal if the transient had typical cardiac Ca^2+^ transient morphology (i.e. rapid upstroke and decay kinetics), no oscillations of the diastolic Ca^2+^ signal, and no obvious spontaneous Ca^2+^ release between transients (Fig. 2c-i, ii). A peak was labeled as abnormal if any of above criteria was not met (Fig. 2c-iii-vi). A cell was labeled as normal if all of the peaks within the cell were normal and of consistent amplitudes and rhythmicity (Fig. 2c-i). A cell was labeled as abnormal if any of above criteria was not met (Fig. 2c-ii-vi).

### Peak detection

To detect the peaks of Ca^2+^ transient signals, we improved the analytical method proposed by Juhola et al*.*^14^*.* Specifically, for each Ca^2+^ transient signal, the first derivative values of signal intensities at the observed timeframe points are first calculated by using the Trapezium rule^16^. Second, a sequential screening strategy is taken to identify the starting, maximum, and ending timeframe points for all peaks presented in the signal. That is, starting from the initial timeframe point or the timeframe point right after the ending of the previous peak, the next timeframe point with first derivative value greater than a pre-defined threshold t_up_ (default 30) is considered as the beginning (i.e., peak left) timeframe point of the current peak. Starting from the peak left, first derivative values should be positive before peak maximum point while negative after peak maximum. Thus, the first timeframe point after the peak left point with a negative derivative value is taken as the maximum timeframe point of the current peak (i.e., peak maximum). The first timeframe point after peak maximum with a positive derivative value whose absolute value is greater than a pre-defined threshold rt_up_ (default 2; to get around possible noisy signal fluctuations) is taken as the end of the current peak (i.e., peak right). The default value for t_up_ is set as used by Juhola et al*.*^14^, and the default value for rt_up_ is determined based on our experiments. In particular, taking rt_up_ = 0 is equivalent as taking the first timeframe point after peak maximum with a positive first derivative value as peak end.

To avoid the identification of a partial or noisy first peak within a signal, we exclude the first peak that is asymmetric with left amplitude less than 50% of the right amplitude and intensity value < 5. We also exclude noisy peaks with peak amplitudes less than 15% of the maximum amplitude within the signal. To ensure that our detected peaks are valid with minimal noise or partial peaks, signals with no peak or a single peak are excluded from our analyses.

### Peak variable quantification

**Figure 3:**
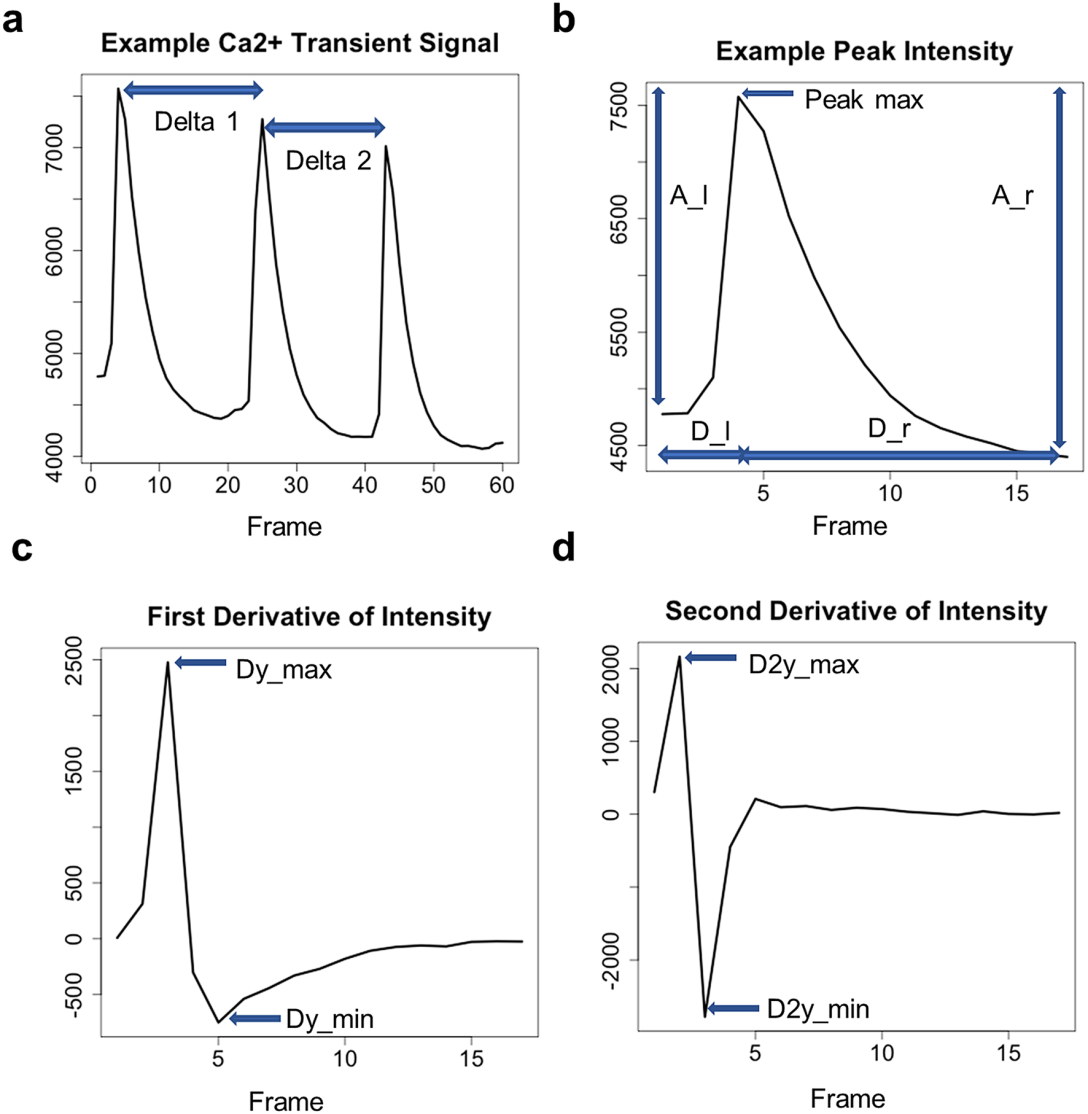
An example of Ca^2+^ transient signal, peak, its first derivative, second derivative, and peak-level variables. Out of 14 peak-level variables, 10 are indicated: (**a**) delta, (**b**) peak left amplitude (A_l), peak right amplitude (A_r), left peak duration (D_l), right peak duration (D_r), (**c**) maximum value of left side first derivative (Dy_max), absolute minimum of right side first derivative (Dy_min), (**d**) maximum of right side second derivative (D2y_max), and absolute minimum of right side second derivative (D2y_min). The peak variables extracted were used for peak status prediction via SVM modeling.

Fourteen peak variables are quantified after peak detection (Fig. 3) and then used for peak abnormality assessment by both analytical and SVM methods.

The 14 peak variables are as follows: peak left amplitude (A_l), peak right amplitude (A_r), amplitude difference between A_l and A_r (A_d), duration from peak left to peak max (D_l), duration from peak max to peak right (D_r), maximum first derivative value from peak left to peak max (Dy_max), absolute minimum first derivative value from peak max to peak right (Dy_min) maximum second derivative value from peak left to peak max (D2y_max), absolute minimum second derivative value from peak max to peak right (D2y_min), peak area under the intensity curve from peak left to peak right (R), duration from the previous peak max to current peak max (delta, i.e., peak distance), duration from peak left to Dy_max (delta_l2Dymax), duration from peak max to Dy_min (delta_m2Dymin), and median of delta values within a signal (Peak_distance_median). These quantified peak variables are used for training peak-level SVM classifier and subsequent cell-level SVM classifier.

### Peak abnormality assessment by improved analytical algorithm

Here, peak max amplitude and min amplitude respectively refer to the maximum and minimum of A_l and A_r. In addition to peak amplitudes and asymmetry as considered by previous method^14^, our improved Analytical Algorithm also considers irregular phase to assess peak normality based on peak distances (delta) within one signal.

We first assess peak normality with respect to peak amplitudes. That is, the first peak will be labeled as abnormal if the peak max amplitude is less than 50% of the average peak max amplitude within the same signal. Peaks other than the first one will be labeled as abnormal if the preceding peak is abnormal and the peak max amplitude is less than 50% of the average peak max amplitude within the same signal, or if the peak amplitude is less than 50% of the preceding normal peak. Second, a peak with normal amplitude characteristics will be labeled as abnormal when the peak min amplitude is less than 85% of the peak max amplitude (i.e., asymmetric). Last, irregular phase assessment will be considered. A symmetric peak with normal amplitude but distance from previous peak to current peak (delta; except for the first peak) greater than 90% of the median delta within the same signal (i.e., irregular phase) will be labeled as abnormal. All thresholds are chosen based on our experimental training data and can be adjusted according to new data characteristics.

### Train peak-level SVM classifier

To employ expert peak assessments and peak characteristics of training data, we train a peak-level SVM classifier to predict peak normality status, taking expert peak assessments as outcome and these 14 peak variables as described in previous subsection as predictive features. To avoid the issue of overfitting for accuracy assessment with training data, we take the LOOCV approach^17^ to fit peak-level SVM classifiers and make predictions for all samples in the training dataset. In particular, peaks within a signal are taken as test data and a corresponding peak-level SVM classifier is trained using peaks from all other samples, which is iterated for all signals to obtain predictions of all peaks. The peak normality predictions by the LOOCV approach will then be used to train the follow-up cell-level SVM classifier.

### Train cell-level SVM classifier

The cell normality labels based on peak normality assessments obtained by our improved Analytical Algorithm and SVM-LOOCV approach are considered as cell variables. We consider additional cell variables as follows: proportion of abnormal peaks per signal (prop_abnormal), variance of peak amplitude per signal (var_A), variance of peak distances per signal (var_delta), and variance of peak areas per signal (var_R). These cell variables are centered and standardized and then used as predictive features to train a cell-level SVM classifier to predict cell abnormality, where outcomes are taken as human-expert assessments about cell normality. This trained cell-level SVM classifier can then be used to predict cell normality for additional independent signals.

### Application studies

To validate the above described pipeline (Fig. 1) for analyzing Ca^2+^ transient data, we applied the pipeline to study the Ca^2+^ transient data of 254 cells generated in our lab. In particular, we took 200 cell signals (containing 1893 peaks) as our training data and 54 cell signals (454 peaks) as our test data. We first manually assessed the normality of all of these signals and peaks that were considered as gold standards and taken as outcome variables for training SVM classifiers. Second, by applying our improved Analytical Methods to assess peak normality, we obtained 93.3% accuracy, 91.1% sensitivity, and 95.8% specificity (Table 3). Third, by the SVM-LOOCV approach to assess peak normality, we obtained 92.2% accuracy, 91.8% sensitivity, and 95.3% specificity (Table 3). Cell abnormality assessments based on these two peak assessments were then taken together with other cell variables to train a cell-level classifier. By using the LOOCV approach with our training data, our cell-level SVM classifier obtained 89.9% accuracy, 94.7% sensitivity, and 83.3% specificity for cell assessments (Table 4).

**Table 3:** Peak abnormality assessment accuracy.

Method	Accuracy (%)	Sensitivity (%)	Specificity (%)
Analytical algorithm	93.3	91.1	95.8
SVM-LOOCV	92.2	91.8	95.3

All accuracy metrics were generated by taking expert cell assessments as the truth and considered for 1893 peaks in the training dataset.

**Table 4:** Cell abnormality assessment accuracy.

Dataset	Method	Accuracy (%)	Sensitivity (%)	Specificity (%)
Training data	Analytical algorithm	87.5	90.4	83.5
	SVM-LOOCV	89.9	94.7	83.3
Test data	Analytical algorithm	83.3	83.3	83.3
	SVM	87.0	88.9	83.3

All accuracy metrics were generated by taking expert cell assessments as the truth and considered for 200 cells in the training data and 54 cells in the test dataset.

With the cell-level SVM classifier trained by using our training data, we then validated the accuracy of cell abnormality assessment with 54 additional test cells. To begin with, by using our pipeline, the Ca^2+^ transient peaks in the test dataset were identified, and peak-level variables were quantified, followed by analytical algorithm peak status assessment. Then, the peak-level SVM classifier trained using our training data produced peak status prediction for each identified peak in the test data. Cell status assessments based on these two peak assessments were used together with other cell variables to predict the final cell normality status by using the trained cell-level SVM model from our training data. As a result, we obtained 87.0% accuracy, 88.9% sensitivity, and 83.3% specificity (Table 4). Compared to the cell abnormality assessments by existing Analytical Algorithm (83.3% accuracy, 83.3% sensitivity, and 83.3% specificity), our SVM approach obtained higher sensitivity and accuracy for borrowing strength across all peaks and signals by SVM method.

**Figure 4:**
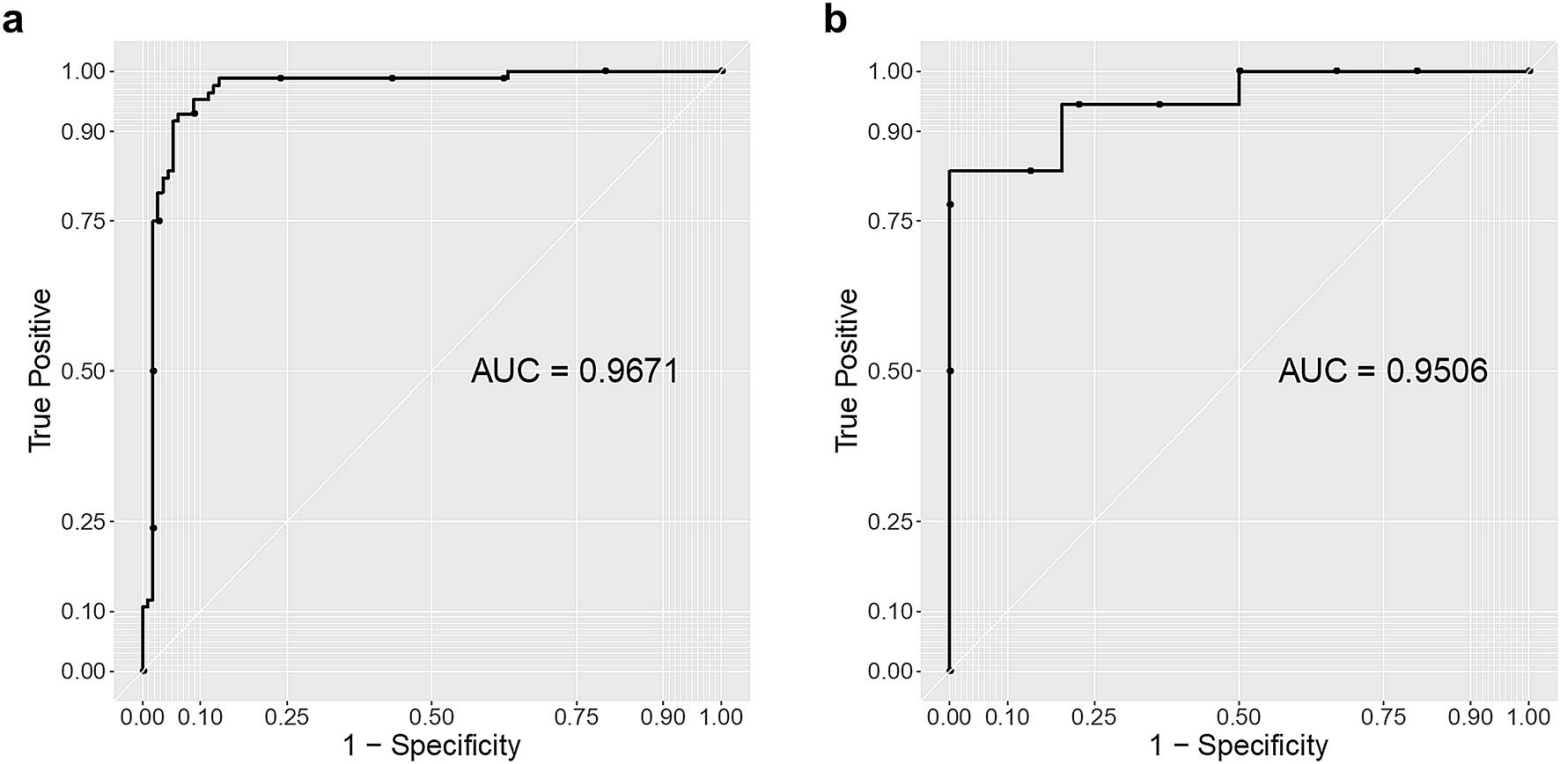
ROC curve plot. (**a**) Training data ROC curve plot. (**b**) Test data ROC curve plot.

In addition, we constructed a receiver-operating curve (ROC)^18^ for both training data and test data based on the classification outcomes of each cell signal by using our trained SVM classifier. As shown in Fig. 4, our trained SVM classifier showed excellent results, with area under the curve (AUC)^18^ of 0.97 and 0.95 for the training and test dataset, respectively. The AUC is the probability that a classifier will rank a randomly chosen abnormal cell higher than a randomly chosen normal cell (assuming 'abnormal' ranks higher than 'normal')^18^.

## Discussion

In this study, we develop an automatic pipeline for assessing the normality of hiPSC-CM Ca^2+^ transient signals, an otherwise labor-intensive and time-consuming phenotypic analysis for CMs. Specifically, we improve the existing Analytical Algorithm^14^ by accounting for irregular phases within signals and employ the advanced machine learning SVM method for peak and cell abnormality prediction. We also validate our approach of using advanced machine learning SVM method in this pipeline by using training and test hiPSC-CM Ca^2+^ transient signals generated by our lab. With independent test data, we demonstrate that our SVM approach obtained 87.0% accuracy (versus 83.3% accuracy obtained by Analytical Algorithm).

Our results show the advantages of learning normal and abnormal characteristics across multiple peaks and cells as well as employing the valuable human-expert assessments of training data. Although our improved Analytical Algorithm yielded excellent peak assessment accuracy of 93.3% with our training data, its cell-level assessment accuracy was 87.5% with our training data and 83.3% with our test data. The decent peak abnormality assessment accuracy by Analytical Algorithm is probably because the Analytical Algorithm is developed to mimic human-expert assessment. In contrast, our cell-level SVM classifier obtained accuracy 89.9% with our training data by the LOOCV approach and accuracy 87.0% with our test data. The relatively lower accuracy for signal abnormality assessment is likely because the Analytical Algorithm fails to account for abnormality due to abnormal characteristics of multiple peaks and signals such as signals with irregular phases.

Automatic identification of Ca^2+^ transients can overcome limitation of traditional manual assessment. Manual signal abnormality assessment is difficult since recordings are often short, contain small number of peaks, with varying morphologies of signals and peaks within them. Abnormal cell signals are often difficult to be identified consistently by multiple human-experts. This may be due to the fact that (1) there are peaks of small amplitude that are borderline noise, (2) the nature of the cell signal morphology renders it difficult to exactly characterize, for instance due to continuously decreasing fluorescence intensity, among other reasons.

Our signal classification results are already applicable for current use. Its potential is even larger as more data can be fed into the training set with ease. By incorporating more data collected and analyzed by different Ca^2+^ transient experts, we expect our model to be better modified for more nuanced prediction of novel Ca^2+^ transient data. Various other types of Ca^2+^ transient signals can be assessed by a human expert to further modify the prediction model based on any potential need of any user.

Currently, our machine learning SVM classifiers have been trained using hiPSC-CMs derived from two different strains of stem cells—SCVI-273 and IMR-90—which produce very similar Ca^2+^ transient signals. This could render the machine learning model biased toward certain signal patterns. As we accumulate more data, we expect to see further improvements in the overall accuracy and efficiency of our proposed machine learning method. In addition, training the model with various other hiPSC-CMs derived from different cell lines including disease cell lines (see, for example, Juhola et al*.*^19^) at multiple differentiation stages will significantly improve the generalizability of our machine learning method in such a way that will allow us to capture the underlying essence of seemingly different patterns of Ca^2+^ transient signals among CMs of different sources. This will, in turn, enhance its ability to be used as a scalable tool for analyzing high-throughput CM data for various purposes, such as drug screening. As our machine learning model incorporates more diverse sets of data, we anticipate its usage to evolve as well: from an aide for a busy human-expert to eventually fully replicating the decision-making of a human-expert on all patterns of Ca^2+^ transient signal, regardless of its origin and experimental procedures employed.

Our Ca^2+^ transient analysis software that implements our automatic pipeline with machine learning SVM method is available in the form of R package for everyone in need of Ca^2+^ transient analysis tool.

## Methods

### Culture of hiPSCs and cardiomyocyte differentiation

Undifferentiated SCVI-273 hiPSCs (Stanford Cardiovascular Institute)^9^ and IMR90 hiPSCs (WiCell Research Institute)^20^ were fed daily on Matrigel-coated plates with mTeSR1 defined medium (Stem Cell Technologies, 85850) and passaged using Versene (Thermo Fisher Scientific, 15040066) when compact colonies reached 90–100% confluence. For CM differentiation, hiPSCs were induced using a growth factor-guided differentiation protocol^21,22^. At the day of induction (day 0), medium was replaced with RPMI 1640 medium supplemented with 2% B27 minus insulin (Thermo Fisher Scientific, A1895601) and 100 ng/ml activin A (R&D Systems, 338-AC-050/CF). After 24 h (day 1), RPMI supplemented with 2% B27 minus insulin was used for 24 h. After 24 h (day 1), activin A was replaced with 10 ng/ml BMP4 (R&D Systems, 314-BP-050/CF), and cells were cultured without any medium change for the next 3 days. From day 4, the growth factor-containing medium was replaced with RPMI supplemented with 2% regular B27 (Thermo Fisher Scientific, 17504044) and the medium was changed every other day. hiPSC-CMs were further enriched by the metabolic selection method using RPMI without glucose (Thermo Fisher Scientific, 11879020) supplemented with 2% B27 and 5 mM lactate from day 11 to 14^23^. Alternatively, enriched hiPSC-CMs were generated by microscale generation of cardiospheres at day 6^24^. Cells were observed under a microscope daily for beating cells, which typically appeared by day 8–10. At day 14, a parallel culture of cells were harvested to determine CM purity before subsequent assessments.

### Ca^2+^ transient assay

Live cell imaging of intracellular Ca^2+^ transient was performed using Fluo-4 AM (Thermo Fisher Scientific, F14202). At differentiation day 18, cells were seeded in a 96-well plate at a low density to acquire single-cell Ca^2+^ transients. At differentiation days 20 to 22, cells were treated with or without arrhythmogenic drugs including TNF-α, ethanol, and melphalan for 3 to 5 days. At differentiation days 23 to 25, cells were acquired for Ca^2+^ transient signals. Beating hiPSC-CMs were incubated with 10 µM Fluo-4 AM for 25 min at 37℃ followed by a 5 min wash with warm 1 × Normal Tyrode solution (148 mM NaCl, 4 mM KCl, 0.5 mM MgCl_2_·6H_2_O, 0.3 mM NaPH_2_O_4_·H_2_O, 5 mM HEPES, 10 mM d-Glucose, 1.8 mM CaCl_2_·H_2_O, pH adjusted to 7.4 with NaOH). Fluorescence images were acquired in 1 × Normal Tyrode’s solution immediately after the wash using ImageXpress Micro XLS System (Molecular Devices) with excitation at 488 nm and emission at 515–600 nm at a frequency of 5 frames/sec and 20× magnification for 12 or 32 s. Fluorescence intensity plots from spontaneously beating cells were obtained using MetaXpress software (Molecular Devices) by region of interest measurements.

Ca^2+^ transients from SCVI-273-derived CMs with and without TNF-a treatment and IMR90-derived CMs with and without ethanol treatment were used as training datasets for machine learning algorithm. Ca^2+^ transients from SCVI-273-derived CMs with or without melphalan treatment were used as test datasets for machine learning algorithm.

We note that treatment conditions we tested (TNF-α, ethanol, and melphalan) caused abnormal Ca^2+^ transients (Fig. 2c and Rampoldi et al*.*^25^) with patterns similar to those observed in patient-derived hiPSC-CMs (e.g., catecholaminergic polymorphic ventricular tachycardia^8^). We also note that the patterns of Ca^2+^ transients at days 23–25 observed in this study were similar to those from cells of 30 ± 2 days old^8^.

### R-package

An R-package for the Ca^2+^ transient analysis described in this study, called SVMCaT, is available on the Github website (https://github.com/hyunmhwang/SVMCaT).

## Supplementary information

Supplementary Information.
