# Supplementary material for: Machine learning identifies abnormal Ca2+transients in human induced pluripotent stem cell-derived cardiomyocytes
Source: Sci Rep. 2020 Oct 12;10:16977. doi: 10.1038/s41598-020-73801-x (PMC7550597; doi:10.1038/s41598-020-73801-x)
Supplement: Supplementary file 1 — Supplementary Information. [file 41598_2020_73801_MOESM1_ESM.pdf]

Supplementary Information

Machine learning identifies abnormal Ca^{2+} transients in human induced pluripotent stem cell-derived cardiomyocytes

Hyun Hwang, Rui Liu, Joshua T. Maxwell, Jingjing Yang, Chunhui Xu

Table S1. Classification of peaks and cells: SVM algorithm vs. expert

Category	Classification	Expert Normal	Expert Abnormal
Peaks (n=454)	SVM Normal	70.3%	1.3%
	SVM Abnormal	11.7%	16.7%
Cells (n=54)	SVM Normal	27.8%	7.4%
	SVM Abnormal	5.6%	59.2%
